# Characteristics and Outcome in Non-Puerperal Uterine Inversion

**DOI:** 10.7759/cureus.13345

**Published:** 2021-02-15

**Authors:** Assaad Kesrouani, Emilia Cortbaoui, Abir Khaddage, Michel Ghossein, Elie Nemr

**Affiliations:** 1 Obstetrics and Gynecology, Saint Joseph University, Beirut, LBN; 2 Pathology, Saint Joseph University, Beirut, LBN; 3 Radiology, Saint Joseph University, Beirut, LBN; 4 Urology, Saint Joseph University, Beirut, LBN

**Keywords:** menorragia, non-puerperal uterine inversion, mri, ultrasound, leiomyoma

## Abstract

Chronic non-puerperal uterine inversion is rare and usually associated with uterine pathology with a diagnosis that is challenging. We present the case of a 47-year-old para 4 Caucasian woman with a history of polyfibromatous uterus who was admitted for severe vaginal bleeding for the past 48 hours associated with hemodynamical instability and was refusing any surgery. Pelvic MRI showed the uterus presenting an unusual appearance with a highly vascularized intracavitary leiomyoma protruding through the cervix. Upon deterioration of her status despite an optimal blood transfusion, resuscitation and anti-fibrinolytic treatment, she accepted total abdominal hysterectomy. The diagnosis of uterine inversion was made intraoperatively and confirmed on histopathologic examination. It revealed two side-by-side benign fundal leiomyomas which had collapsed the fundus and protruded partly from the cervix. Non-puerperal chronic inversion of the uterus is rare, and its diagnosis should be based on ultrasound, pelvic MRI and a high index of suspicion, allowing rapid diagnosis and treatment and thus decreasing patient morbidity and mortality.

## Introduction

Uterine inversion is the folding of the fundus into the uterine cavity through the cervix. It is usually a serious postpartum complication leading to massive hemorrhage which generally occurs in the third stage of labor. Chronic uterine inversion is very rare and is observed at least four weeks after delivery (in an obstetrical setting). About one-sixth of all inversion cases are spontaneous, non-puerperal, and are called chronic or gynecological uterine inversions [1-3]. In some studies, the uterine inversion is further stratified by extent: incomplete (no part of corpus past cervix), complete (inversion extends into the vagina), and prolapsed (protrudes past the introitus). We present a case of a spontaneous chronic uterine inversion that was particularly challenging.

## Case presentation

A 47-year-old Caucasian female, gravida 4 para 4 (two normal deliveries and two cesarean sections), came to the Emergency Department for severe vaginal bleeding in the past 48 hours associated with syncope and hemodynamical instability. She had been complaining of intermittent menometrorrhagia over the past year that was attributed to a polyfibromatous uterus visualized on abdominal ultrasound, but the patient had declined all medical and surgical treatments. Two years prior to this event, she had a non-complicated hysteroscopic myomectomy for a 4 cm intracavitary myoma with no malignancy at the pathology.

**Figure 1:**
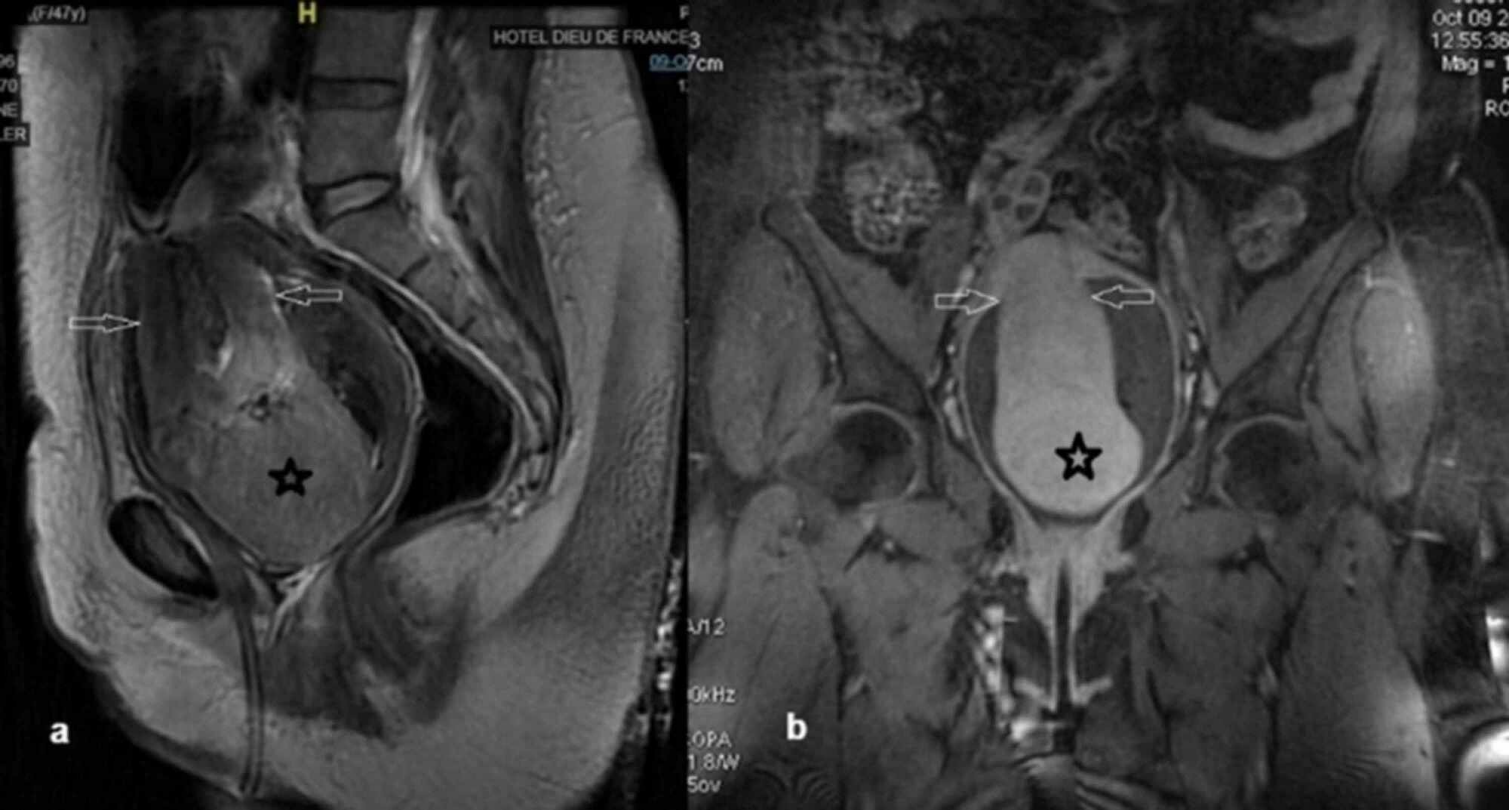
MRI characteristics of the uterine inversion In a: T2-sagittal view, In b: coronal view. The two arrows depict the dumbbell appearance. The myoma is depicted by a *.

**Figure 2:**
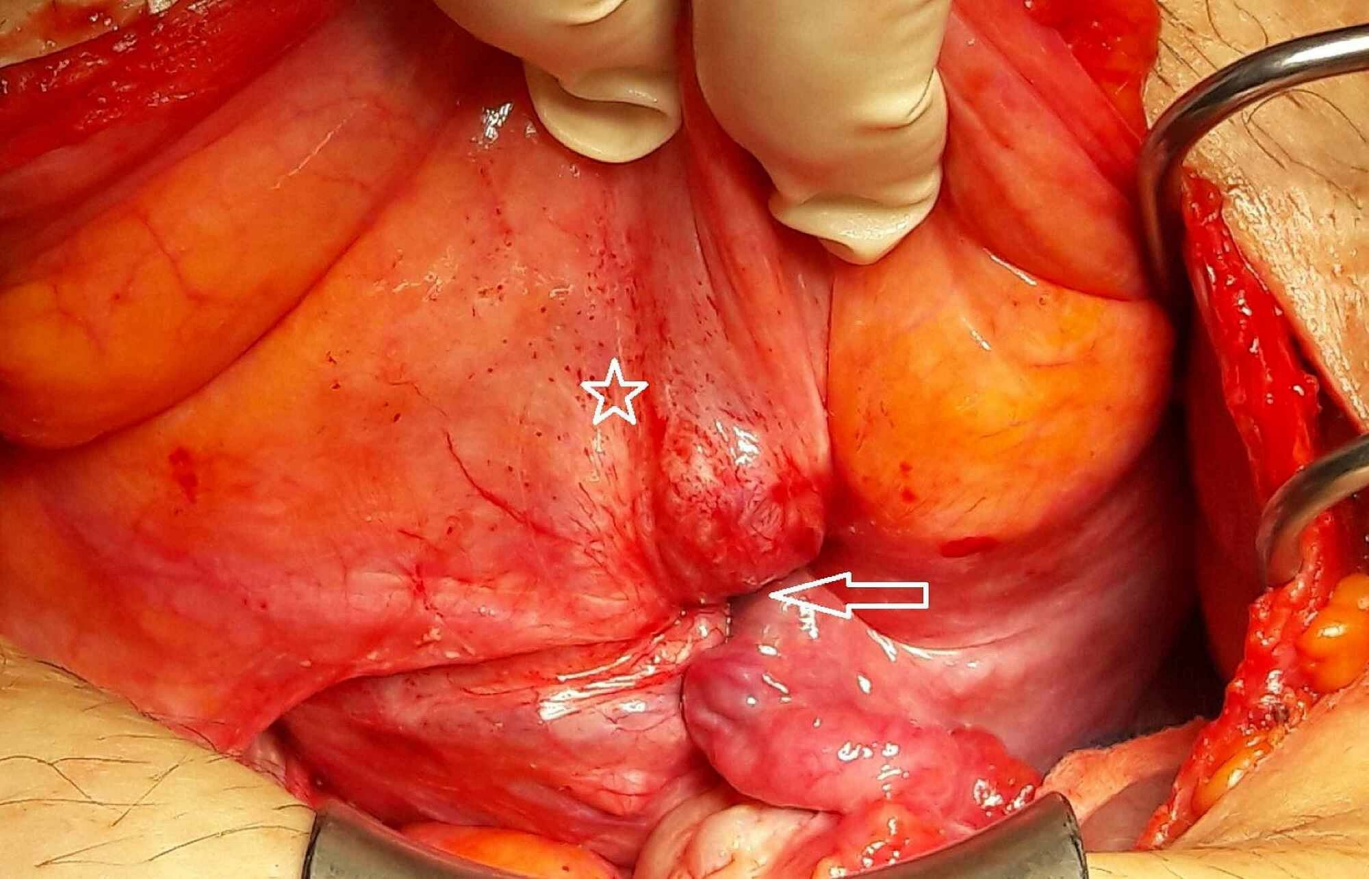
Intraoperative appearance during laparotomy The arrow indicates the area of the fundal prolapse with the left adnexa plunging inside the recess. The bladder is depicted by a *.

**Figure 3:**
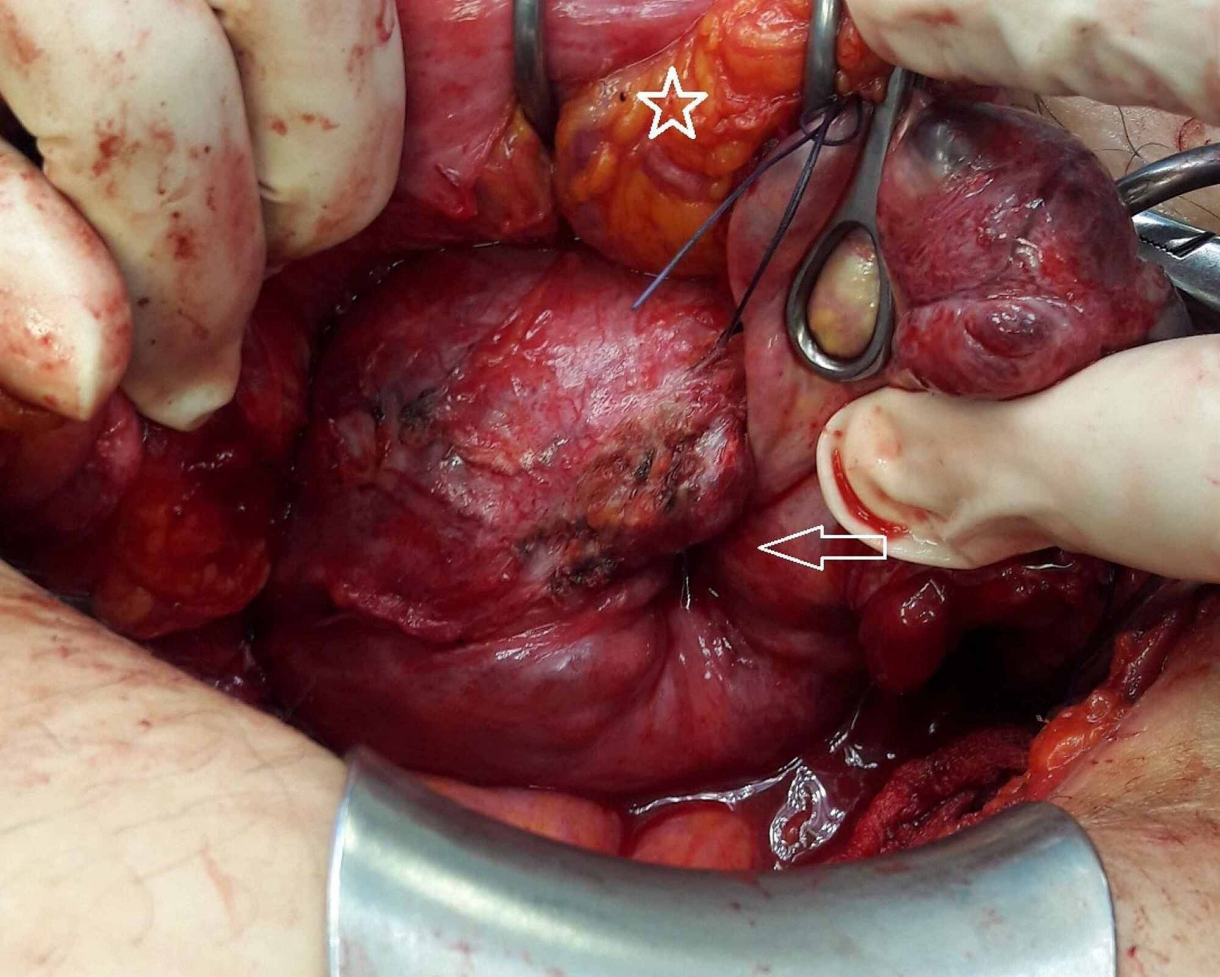
Intraoperative appearance after dissecting the bladder. The arrow indicates the area of the fundal prolapse. The bladder is depicted by a *.

Upon admission, the patient appeared pale but responsive with a pulse of 105 bpm, blood pressure (BP) 130/80 mmHg, and a hemoglobin value of 5.6 g/dL. Clinical examination revealed an enlarged uterus with a 6-7 cm firm non-sensitive mass protruding from the cervix. Transfusion was initiated upon the patient's initial refusal of a surgical treatment along with anti-fibrinolytic treatment to stop the bleeding. A pelvic MRI enhanced with gadolinium was interpreted with caution as it showed a 12.8 x 10 x 9.5 cm uterus presenting an unusual bell shape with a highly vascularized 11 x 7.9 x 6.6 cm intracavitary lesion (most probably a leiomyoma) that was protruding through the cervix (Figure 1). Despite adequate medical treatment and receiving six packs of red blood cells, the bleeding couldn’t be stopped and her hemoglobin level remained at 7.3 g/dL. The patient finally consented to a total abdominal hysterectomy after discussing other possible conservative surgical management. The surgery uncovered an enlarged polyfibromatous uterus with a fundus collapsed in the middle creating a gap and grasping the right adnexa. The vagina, on the other hand, was elevated around the uterus creating a turtle neck (Figure 2, 3). Surgery was difficult as the anatomical landmarks were markedly distorted; ureteral dissection was carried out by an experienced urologist to avoid any accidental lesion. Upon completion of the hysterectomy, the uterus unfolded spontaneously revealing the uterine inversion that had distorted its aspect on MRI. The pathology examination, along with a reconstitution of the uterine inversion, revealed that two side-by-side submucosal fundal leiomyomas measuring 5.5 and 2 cm each had collapsed the fundus and protruded partly from the cervix, therefore leading to a chronic, non-puerperal uterine inversion. These leiomyomas had hemorrhagic modifications due to ischemic necrosis; no evidence of malignancy was found.

During her hospitalization, the patient received a total of 10 packed red blood cells (PRBC) and two fresh frozen plasma (FFP). She made a full uneventful recovery and was discharged home on day four.

This case was submitted to our institution's ethics committee for approval and was registered as CEHDF-1137.

## Discussion

Non-puerperal uterine inversion is extremely rare, representing 16% of all uterine inversions [1]. Less than 200 cases are reported in the literature [2].

Uterine inversion can be classified into four stages as described by Salomon et al.: Stage 1: the inverted uterus remains in the uterine cavity; Stage 2: complete inversion of the fundus through the cervix; Stage 3: the inverted fundus protrudes through vulva; and Stage 4: inversion of the uterus and vaginal wall through the vulva [1,4].

The clinical diagnosis of chronic uterine inversion mostly depends on the finding of a mass protruding out of the cervix (for Stages 2-4) and the absence of a uterine body during bimanual examination. The clinical diagnosis of Stage 1 is difficult and requires a high index of suspicion and appropriate imaging. The prominent symptoms are vaginal discharge, irregular uterine bleeding, and pelvic discomfort [1]. Menorrhagia can lead to hypovolemia and transfusion as seen with our patient.

The etiology of the inversion is not clearly defined and it appears to be multifactorial. The following etiologies have been described in the literature: uterine leiomyoma, leiomyosarcoma, rhabdomyosarcoma, endometrial polyps, endometrial carcinoma, and total uterovaginal prolapse [1,2,5]. In addition, a rapid growth of the tumor, a fundal location of the tumor, a thin uterine wall, and dilatation of the cervix can all precipitate the inversion [6]. 

Prolapse and extrusion of a leiomyoma, especially a submucous leiomyoma of the fundus, tends to be the most common factor inciting the inversion in 80-85% of cases, along with other etiologies such as hormone replacement and the presence of increased intra-abdominal pressure [2,6]. A 2018 systematic review of 170 case reports found that benign leiomyomas were the leading cause of chronic uterine inversion (57.2%) followed by leiomyosarcomas at 13.5% [6]. There was no evidence of abnormalities in 9.9% of all cases [6]. A recent literature review concluded that malignancy was present in one-third of the reported cases [7]. This should be discussed with patients when choosing the type of surgical management.

Ultrasound and MRI are important imaging tools that play a major role in the diagnosis of this entity. Ultrasound findings include a depressed longitudinal groove extending from the uterus to the center of the inverted uterus [4]. Lewin et al. reported that in T2-weighted MRI scans, a U-shaped uterine cavity and a thickened and inverted uterine fundus on a sagittal image and a “bull’s-eye” configuration on an axial image are signs indicative of uterine inversion [5]. Takano et al. also reported a case in which a U-shaped uterine cavity was imaged with a T1-weighted dynamic MRI enhanced by gadolinium [3]. However, because of the rarity of this entity, these elements are often hard to interpret. The preoperative diagnosis is, thus, difficult and requires a high index of suspicion [1,2].

Chronic uterine inversion is difficult to treat conservatively and surgery is usually needed. The morbidity and mortality associated with uterine inversion correlate with the degree of hemorrhage and the rapidity of diagnosis and treatment [4]. Chronic uterine inversion is best treated by total hysterectomy which remains the main treatment in 86.8%, abdominal approach in 78.6%, and vaginal approach in 21.4% [6]. However, choosing this recourse will depend on the patient's future fertility desire. Conservative abdominal procedures that have been described to correct the inversion include: (a) serial clamping and upward traction on the round ligaments to elevate the fundus (Huntington procedure) and (b) vertical incision along the posterior wall of the uterus in the area of the constriction and then manual repositioning of the fundus (Haultain procedure). Conservative vaginal approaches, including the Kustner and Spinelli procedures, are also reported [1,4,6]. Robotic and laparoscopic surgeries and abdominal cerclage have been recently used to correct chronic uterine inversion [4,8].

## Conclusions

Spontaneous non-puerperal chronic uterine inversion is an exceptional and life-threatening disease that most gynecologists will rarely encounter in their career. It is usually associated with uterine pathology. Careful examination, imaging studies such as pelvic ultrasound and MRI coupled with a high index of suspicion will yield correct diagnosis and treatment that would help to decrease patient morbidity and mortality.
